# Supplementary material for: Inhibition of the neuromuscular acetylcholine receptor with atracurium activates FOXO/DAF‐16‐induced longevity
Source: Aging Cell. 2021 Jul 6;20(8):e13381. doi: 10.1111/acel.13381 (PMC8373276; doi:10.1111/acel.13381)
Supplement: Supplementary file 2 — Supplementary Material [file ACEL-20-e13381-s003.docx]

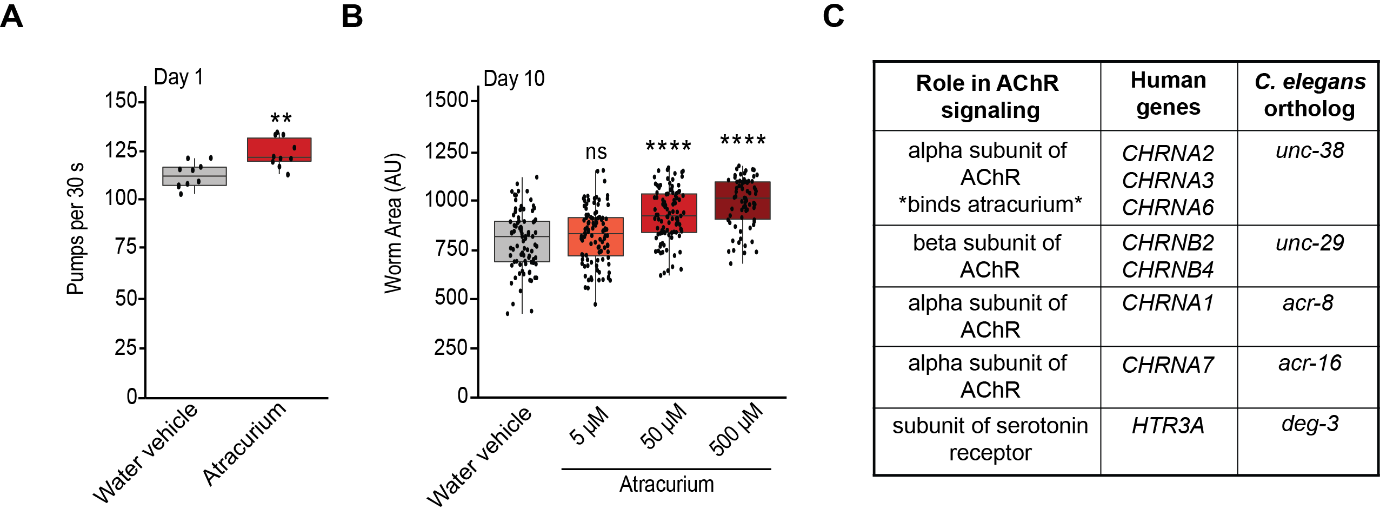


**Figure S1: Atracurium extends healthspan and lifespan by antagonizing the neuromuscular acetylcholine receptor.**

A) Pharyngeal pumping at day 1 of adulthood in control and atracurium (50 μM) treated N2 worms. Despite its clinical function as an anesthetic muscle relaxant, atracurium does not reduce pumping. For each condition, n=10 worms. Statistical significance is determined by unpaired t-test. B) Body size of atracurium-treated N2 worms at day 10 of adulthood. Increased concentration of atracurium causes increased body size in a dose-dependent manner (5 μM, 50 μM , 500 μM). For each condition, n=~70-110 measurements of ~50 worms. Statistical significance is determined by a one-way ANOVA, and represented p-values are each compared to the water vehicle. Healthspan statistics can be found in Table S3 ****p<0.0001, **p<0.01, ns - not significant. C) Table describing orthogolous ACh signaling genes in humans and *C. elegans*. In humans, atracurium binds the AChR alpha subunit encoded by the *CHRNA2* gene, *unc-38* in worms.

**
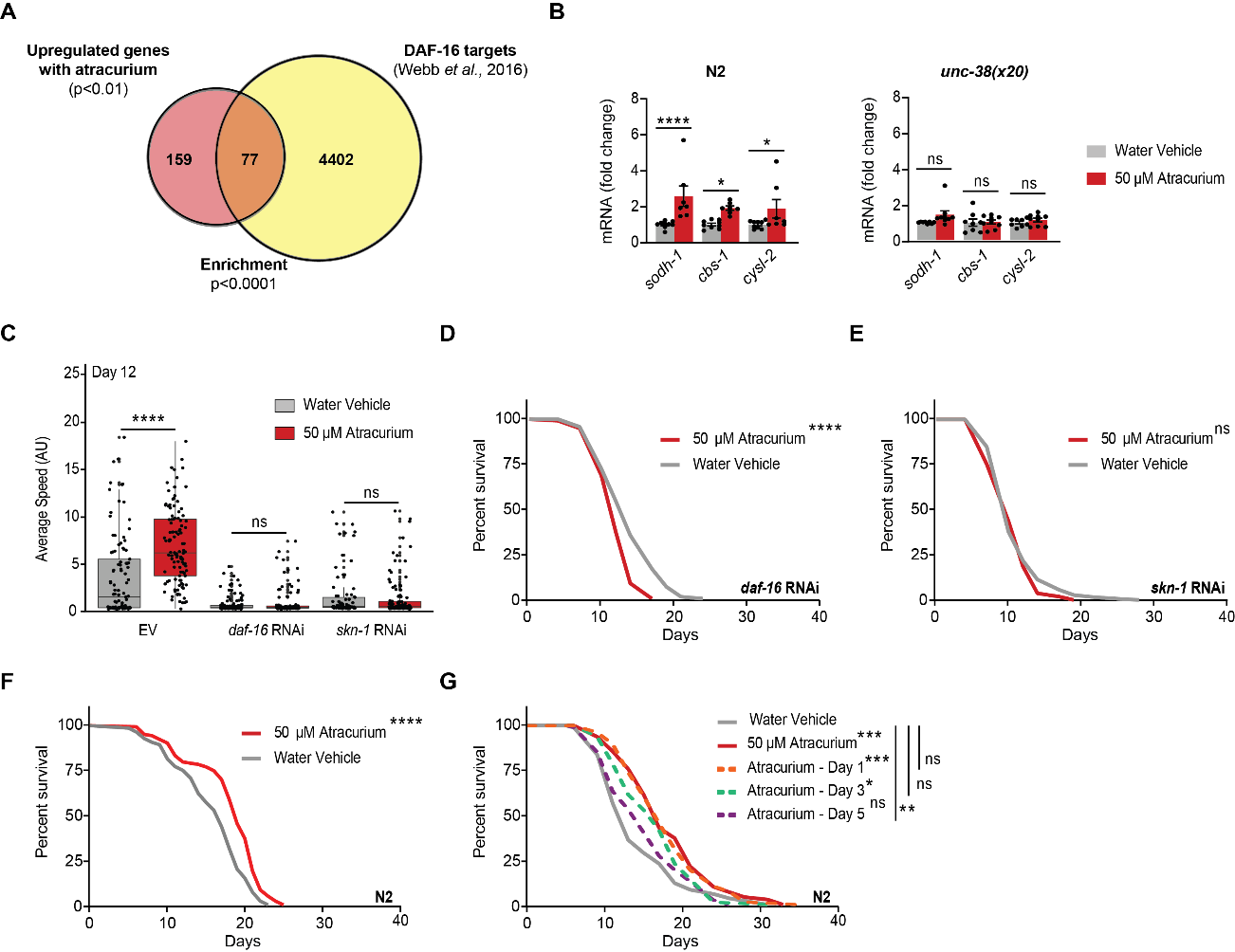
**

**Figure S2: Mechanism by which atracurium extends lifespan**

A) Venn diagram depicting overlap between upregulated genes from the RNA sequencing transcriptomics depicted in Figure 5 and genes upregulated upon *daf-16* overexpression (Webb et al., 2016). The differentially expressed genes with atracurium treatment were significantly enriched for DAF-16 target genes, as determined by a Fisher’s Exact test (p<0.0001). B) The transcript level of DAF-16 regulated genes upregulated with atracurium in Figure 5 in day 1 adult N2 and *unc-38(x20)* mutant worms upon atracurium treatment. The expression levels of all genes were normalized to reference genes *ama-1* and *cdc-42*, and compared with the mean value of untreated worms. Results were pooled from two independent experiments. Statistical significance is determined by two-way ANOVA. C) Mobility of atracurium-treated (50 μM) N2 worms upon empty vector, *daf-16* RNAi and *skn-1* RNAi treatment at day 12 of adulthood. Atracurium improves healthspan in control worms, but this effect is abolished in worms with *daf-16* or *skn-1* knockdown. For each condition, n=~80-110 measurements of ~50 worms. Statistical significance is determined by one-way ANOVA. See Table S3 for healthspan statistics. D) Survival curves showing that atracurium (50 μM) does not extend lifespan in presence of *daf-16* RNAi (atracurium shortens lifespan p<0.0001). E) Survival curves showing that atracurium (50 μM) does not extend lifespan in presence of *skn-1* RNAi. For each lifespan condition, n=120. F) Survival curves showing that atracurium (50 μM) extends lifespan in the absence of 5-fluouridine. Lifespan was measured using the microfluidics system of NemaLife Inc. Statistical comparisons of survival curves are determined by log-rank tests. For each condition, n=~150 worms. G) Survival curves showing that atracurium (50 μM) treatment beginning at L4 developmental stage, and day 1 and day 3 of adulthood extend lifespan, but beginning at day 5 of adulthood does not. For each condition, n=100 worms. Lifespan statistics are determined by log-rank tests. See Table S2 for lifespan statistics. ****p<0.0001, ***p<0.001, **p<0.01, *p<0.5, ns - not significant.

**Table S2: Lifespan Statistics**

| ***C. elegans* strain** | **Treatment** | **Median lifespan (days)** | **% Change** | | **Number animals (died/total)** | | **P – value against control group** |
| --- | --- | --- | --- | --- | --- | --- | --- |
| **Figure 2 Panel A** | | | | | | | |
| Wild Type (N2)* | DMSO vehicle | 16 |  | | 86/100 | |  |
|  | 50 μM zidovudine | 18 | 31.25% | | 76/100 | | <0.0001 |
| Wild Type (N2) | DMSO vehicle | 15 |  | | 64/100 | |  |
|  | 50 μM zidovudine | 18 | 20.00% | | 69/100 | | 0.0024 |
| Wild Type (N2) | DMSO vehicle | 16 |  | | 90/100 | |  |
|  | 50 μM zidovudine | 19 | 18.75% | | 80/100 | | 0.0001 |
| Wild Type (N2) | DMSO vehicle | 11 |  | | 77/100 | |  |
|  | 50 μM zidovudine | 25 | 127.30% | | 76/100 | | <0.0001 |
| **Figure 2 Panel B** | | | | | | | |
| Wild Type (N2)* | water vehicle | 13 |  | | 82/100 | |  |
|  | 50 μM atracurium | 20 | 58.33% | | 81/100 | | <0.0001 |
| Wild Type (N2) | water vehicle | 15 |  | | 72/100 | |  |
|  | 50 μM atracurium | 18 | 20.00% | | 71/100 | | <0.0001 |
| Wild Type (N2) | water vehicle | 13 |  | | 85/100 | |  |
|  | 50 μM atracurium | 16 | 23.08% | | 72/100 | | 0.0055 |
| Wild Type (N2) | water vehicle | 17 |  | | 85/100 | |  |
|  | 50 μM atracurium | 22 | 22.73% | | 76/100 | | 0.0002 |
| Wild Type (N2)^$^ | water vehicle | 16 |  | | 77/100 | |  |
|  | 50 μM atracurium | 22 | 37.50% | | 63/100 | | 0.0004 |
| **Figure 2 Panel C** | | | | | | | |
| *daf-16(mu86)** | DMSO vehicle | 12 |  | | 82/100 | |  |
|  | 50 μM zidovudine | 19 | 58.33% | | 81/100 | | <0.0001 |
| *daf-16(mu86)* | DMSO vehicle | 13 |  | | 85/100 | |  |
|  | 50 μM zidovudine | 18 | 38.46% | | 69/100 | | <0.0001 |
| *daf-16(mu86)* | DMSO vehicle | 10 |  | | 84/100 | |  |
|  | 50 μM zidovudine | 19 | 90.00% | | 81/100 | | <0.0001 |
| **Figure 2 Panel D** | | | | | | | |
| *daf-16(mu86)** | water vehicle | 13 |  | | 82/100 | |  |
|  | 50 μM atracurium | 13 | 0.00% | | 81/100 | | 0.0364 |
| *daf-16(mu86)* | water vehicle | 12 |  | | 85/100 | |  |
|  | 50 μM atracurium | 15 | 25.00% | | 69/100 | | 0.0382 |
| *daf-16(mu86)* | water vehicle | 12 |  | | 64/100 | |  |
|  | 50 μM atracurium | 14 | 7.69% | | 81/100 | | <0.0001 |
| **Figure 2 Panel F** | | | | | | | |
| Wild Type (N2)* | water vehicle | 13 |  | | 84/100 | |  |
|  | 5 μM atracurium | 15 | 15.39% | | 76/100 | | 0.0257 |
|  | 50 μM atracurium | 17 | 30.77% | | 71/100 | | 0.0001 |
|  | 500 μM atracurium | 19 | 46.15% | | 77/100 | | <0.0001 |
| Wild Type (N2) | water vehicle | 15 |  | | 85/100 | |  |
|  | 5 μM atracurium | 22 | 46.67% | | 79/100 | | <0.0001 |
|  | 50 μM atracurium | 19 | 26.67% | | 80/100 | | 0.0142 |
|  | 500 μM atracurium | 22 | 46.67% | | 81/100 | | <0.0001 |
| **Figure 3 Panel C** | | | | | | | |
| Wild Type (N2)* | water vehicle | 17 |  | | 85/100 | |  |
|  | 50 μM atracurium | 22 | 22.73% | | 76/100 | | 0.0002 |
| Wild Type (N2) | water vehicle | 13 |  | | 82/100 | |  |
|  | 50 μM atracurium | 20 | 58.33% | | 81/100 | | <0.0001 |
| Wild Type (N2) | water vehicle | 15 |  | | 72/100 | |  |
|  | 50 μM atracurium | 18 | 20.00% | | 71/100 | | <0.0001 |
| Wild Type (N2) | water vehicle | 13 |  | | 85/100 | |  |
|  | 50 μM atracurium | 16 | 23.08% | | 72/100 | | 0.0055 |
| Wild Type (N2)^$^ | water vehicle | 16 |  | | 77/100 | |  |
|  | 50 μM atracurium | 22 | 37.50% | | 63/100 | | 0.0004 |
| **Figure 3 Panel D** | | | | | | | |
| *unc-38(x20)** | water vehicle | 19 |  | | 66/100 | |  |
|  | 50 μM atracurium | 19 | 0.00% | | 79/100 | | 0.1718 |
| *unc-38(x20)* | water vehicle | 23 |  | | 81/100 | |  |
|  | 50 μM atracurium | 21 | -8.70% | | 78/100 | | 0.0878 |
| *unc-38(x20)^$^* | water vehicle | 25 |  | | 41/100 | |  |
|  | 50 μM atracurium | 25 | 0.00% | | 39/100 | | 0.0878 |
| **Figure 3 Panel E** | | | | | | | |
| *unc-38(e264)** | water vehicle | 15 |  | | 83/100 | |  |
|  | 50 μM atracurium | 11 | -26.67% | | 82/100 | | <0.0001 |
| *unc-38(e264)* | water vehicle | 13 |  | | 88/100 | |  |
|  | 50 μM atracurium | 13 | 0.00% | | 68/100 | | <0.0001 |
| *unc-38(e264)^$^* | water vehicle | 16 |  | | 81/100 | |  |
|  | 50 μM atracurium | 14 | -14.29% | | 66/100 | | <0.0001 |
| **Figure 4 Panel D** | | | | | | | |
| Wild Type (N2)* | empty vector (EV) +  water vehicle | 19 |  | | 103/120 | |  |
|  | empty vector (EV) +  50 μM atracurium | 21 | 10.52% | | 90/120 | | 0.0212 |
|  | *unc-38* RNAi +  water vehicle | 21 | 10.52% | | 73/120 | | 0.0003 |
|  | *unc-38* RNAi +  50 μM atracurium | 19 | 0.00% | | 95/120 | | <0.0001 |
| Wild Type (N2) | empty vector (EV) + water vehicle | 17 |  | | 89/110 | |  |
|  | empty vector (EV) +  50 μM atracurium | 19 | 11.77% | | 86/110 | | 0.0250 |
|  | *unc-38* RNAi + water vehicle | 21 | 23.53% | | 69/110 | | 0.0028 |
|  | *unc-38* RNAi +  50 μM atracurium | 19 | 11.77% | | 87/110 | | 0.0042 |
| **Figure S2 Panel D** | | | | | | | |
| Wild Type (N2)* | *daf-16* RNAi +  water vehicle | 14 |  | | 103/120 | |  |
|  | *daf-16* RNAi +  50 μM atracurium | 12 | -14.29% | | 107/120 | | <0.0001 |
| Wild Type (N2) | *daf-16* RNAi +  water vehicle | 13 |  | | 92/110 | |  |
|  | *daf-16* RNAi +  50 μM atracurium | 13 | 0.00% | | 90/110 | | 0.0002 |
| **Figure S2 Panel E** | | | | | | | |
| Wild Type (N2)* | *skn-1* RNAi +  water vehicle | 10 |  | | 109/120 | |  |
|  | *skn-1* RNAi +  50 μM atracurium | 10 | 0.00% | | 110/120 | | 0.1176 |
| Wild Type (N2) | *skn-1* RNAi +  water vehicle | 13 |  | | 94/110 | |  |
|  | *skn-1* RNAi +  50 μM atracurium | 11 | -15.39% | | 98/110 | | 0.1258 |
| **Figure S2 Panel F (no 5FU)** | | | | | | | |
| Wild Type (N2)* | water vehicle | 17 | |  | | 140/140 |  |
|  | 50 μM atracurium | 19 | | 11.77% | | 143/143 | <0.0001 |
| Wild Type (N2) | water vehicle | 16 | |  | | 132/132 |  |
|  | 50 μM atracurium | 17 | | 6.25% | | 122/134 | 0.0102 |
| Wild Type (N2) | water vehicle | 15 | |  | | 77/77 |  |
|  | 50 μM atracurium | 17 | | 13.33% | | 137/143 | 0.0012 |
| **Figure S2 Panel G** | | | | | | | |
| Wild Type (N2)* | water vehicle | 13 |  | | 84/100 | |  |
|  | 50 μM atracurium | 17 | 30.77% | | 71/100 | | 0.0001 |
|  | 50 μM atracurium – from day 1 adulthood | 17 | 30.77% | | 72/100 | | 0.0002 |
|  | 50 μM atracurium – from day 3 adulthood | 17 | 30.77% | | 71/100 | | 0.0189 |
|  | 50 μM atracurium – from day 5 adulthood | 15 | 15.38% | | 80/100 | | 0.4312 |
| Wild Type (N2) | water vehicle | 15 |  | | 85/100 | |  |
|  | 50 μM atracurium | 19 | 26.67% | | 80/100 | | 0.0142 |
|  | 50 μM atracurium – from day 1 adulthood | 22 | 46.67% | | 78/100 | | <0.0001 |
|  | 50 μM atracurium – from day 3 adulthood | 19 | 26.67% | | 73/100 | | 0.0026 |
|  | 50 μM atracurium – from day 5 adulthood | 17 | 13.33% | | 74/100 | | 0.3649 |
| **Not shown** | | | | | | | |
| Wild Type (N2) | DMSO vehicle | 15 |  | | 64/100 | |  |
|  | 50 μM sildenafil | 15 | 0.00% | | 82/100 | | 0.4184 |
| Wild Type (N2) | DMSO vehicle | 16 |  | | 90/100 | |  |
|  | 50 μM sildenafil | 14 | -12.50% | | 90/100 | | 0.169 |
| Wild Type (N2) | DMSO vehicle | 16 |  | | 86/100 | |  |
|  | 50 μM sildenafil | 13 | -18.75% | | 77/100 | | 0.0807 |

*experiment represented in figure

^$^experiment was stopped amid the COVID-19 crisis, though all conditions reached the point of 50% viability

**Table S6: Primers used for HL1 mouse cardiomyocyte qPCR**

| **Gene** | **Forward (sequence 5’→3’)** | **Reverse (sequence 5’→3’)** |
| --- | --- | --- |
| **Reference gene** |  |  |
| *Gapdh* | GGTGCTGAGTATGTCGTGGA | GACGTGGTGGTTGACGAATC |
| **Target genes** |  |  |
| *Cat* | TTGACAGAGAGCGGATTCT | GCAAGCTAAGAGGTGTCAGT |
| *Ccng2* | AAAGGGCTGATCTTGATGGA | TTTGGCTTTGTGGACAGGAC |
| *Bcl6* | CTGCAGATGGAGCATGTTGT | AGCACCTCTTGTTATACGGT |
| *Scp2* | CCTGATATGGCAAAGGAAGC | CAAACCCTGACTGACCGTAA |
| *Foxo3* | TTCAAGGATAAGGGCGACAG | TTCATGTGGTTCTCGGCTCC |
| *Hspa1b* | TGGTGCTGACGAAGATGAAG | TTGCACGAGTAGAAGCTGGA |
| *Gadd45a* | GCTCAACGTAGACCCCGATA | GACGACGATGACCTCTTGCT |

**Table S7: Primers used for *C. elegans* qPCR**

| **Gene** | **Forward (sequence 5’→3’)** | **Reverse (sequence 5’→3’)** |
| --- | --- | --- |
| **Reference gene** |  |  |
| *cdc-42* | TCGACAATTACGCCGTCACA | AGGCACCCATTTTTCTCGGA |
| *ama-1* | AAGAAGGTCGCAGGTGGATG | GTGGTGGGACTGGAAGTACG |
| **Target genes** |  |  |
| *sodh-1* | ACACGAAGGAGCTGGAAGTG | TTCCGTGATCCATTGCGACA |
| *cbs-1* | AGACCGTACCGCTGACAAAG | GACTCCAACAACGCGAACAC |
| *cysl-2* | GAACTTGCACTGGAGTCGGA | AACTGTTTCATGTTGGCGGC |
